# The Role of Soluble Uric Acid in Modulating Autophagy Flux and Inflammasome Activation during Bacterial Infection in Macrophages

**DOI:** 10.3390/biomedicines8120598

**Published:** 2020-12-12

**Authors:** Duha Al-Awad, Nada Al-Emadi, Marawan Abu-Madi, Asmaa A. Al-Thani, Susu M. Zughaier

**Affiliations:** 1 Biomedical Science Department, College of Health Sciences, Qatar University, P.O. Box 2713 Doha, Qatar; duha_awad@hotmail.com (D.A.-A.); Nada.Al-Emadi@qu.edu.qa (N.A.-E.); abumadi@qu.edu.qa (M.A.-M.); aaja@qu.edu.qa (A.A.A.-T.); 2 Biomedical and Pharmaceutical Research Unit, QU Health, Qatar University, P.O. Box 2713 Doha, Qatar; 3 Biomedical Research Center, Qatar University, P.O. Box 2713 Doha, Qatar; 4 College of Medicine, QU Health, Qatar University, P.O. Box 2713 Doha, Qatar

**Keywords:** hyperuricemia, uric acid, inflammation, bacterial infection, autophagy flux, macrophages, inflammasome, IL-1β

## Abstract

Autophagy is a homeostatic process that regulates and recycles intracellular structures and is a host defense mechanism that facilitates bacterial clearance. Uric acid in plasma is a major antioxidant but in certain conditions acts as an inflammatory danger signal. The aim of this study is to investigate the effect of soluble uric acid on autophagy and the inflammatory responses in macrophages during bacterial infection. Herein, we employed murine RAW264.7 macrophages that express uricase enzyme and human THP-1 cells that are uricase-deficient. Three different strains of *Staphylococcus aureus* and two different strains of *Klebsiella pneumoniae* were used to infect macrophages in presence and absence of soluble uric acid. We found that soluble uric acid enhanced autophagy flux in infected macrophages. We observed that IL-1β increased during bacterial infection but decreased when macrophages were co-stimulated with bacteria and uric acid. In contrast to IL-1β, soluble uric acid did not affect TNFα release and there were no dramatic differences when macrophages were infected with *S. aureus* or *K. pneumoniae*. In conclusion, uric acid enhances autophagy flux during bacterial infection, consequently reducing inflammasome activation in macrophages. Understanding the effect of uric acid on the interplay between autophagy and inflammation will facilitate therapeutic design.

## 1. Introduction

Hyperuricemia describes the presence of abnormally high levels of uric acid in the blood stream (>5.5 mg/dL in children and young adults, >6mg/dL in women, and >7mg/dL in men). Uric acid is a waste product released during purine catabolism [1,2]. Accumulation of uric acid is due to increased production with unavailability of the degradation enzyme uricase [3]. Humans lack the uricase enzyme, which converts uric acid to a more soluble product, allantoin [4]. Uric acid is released from the cells in the soluble form; however, after exceeding 6.8 mg/dL concentration in a solution, monosodium urate crystals (MSU) begin forming with the aid of other enhancing factors such as low temperatures and acidic environments [5,6,7]. Uric acid has antioxidant properties when present in body fluids at normal physiological levels. In contrast, at higher concentrations, it appears to become pro-inflammatory [8,9]. Uric acid is mostly excreted through kidneys and some through intestine; however, elevated uric acid is implicated in kidney stone formation [10]. Further, phagocytes take up uric acid crystals, leading to proinflammatory response and MSU accumulation in the joints, causing chondrocyte death and, thus, are implicated in the pathogenesis of Gouty arthritis [11].

Hyperuricemia is a pathophysiological condition observed in association with chronic inflammatory diseases such as arthritis, diabetes, cardiovascular, and kidney diseases [1,11]. Hyperuricemia often leads to the formation of monosodium urate (MSU) crystals. These crystals then deposit in different tissues, resulting in inflammation. These events are the hallmark of gout, an inflammatory arthritis that causes severe joint pain [12]. Hyperuricemia does not always lead to the development of gout and could be asymptomatic. However, asymptomatic hyperuricemia has been linked to a condition, such as chronic inflammatory states, kidney disease, cardiovascular disease, and metabolic syndrome [13].

IL-1β is a pro-inflammatory cytokine that plays a critical role during immune responses. The release of IL-1β results in the recruitment and differentiation of proinflammatory cells and the induction of proinflammatory mediators, thereby driving local and systemic immune responses such as tissue damage, neutrophilia, inflammation, and fever [14]. The NLRP3 inflammasome is a major inflammatory protein complex that triggers the release of IL-1β upon activation [15].

Autophagy is a homeostatic process involved in recycling macromolecules within cells. The role of autophagy extends beyond housekeeping. Autophagy is involved in cellular responses to different forms of stressors such as starvation, hypoxia, inflammation, infection, and ER stress [16]. Autophagy contributes to innate immunity by sequestering and degrading invading microorganisms [17]. However, bacterial pathogens evolved mechanisms to evade autophagy [18]. We recently reported that phosphoethanolamine modification on lipid A of *Neisseria gonorrhoeae* reduces autophagy flux in macrophages, which facilitate bacterial survival and promote infection [19]. The link between autophagy induction and proinflammatory cytokines suppression and degradation has been reported in several studies [20,21,22]. Induction of autophagy dampens the immune response through several mechanisms. Controlling the activation of the NLRP3 inflammasome and the subsequent release of IL-1β is perhaps the most prominent of these mechanism [20].

The nucleation of uric acid and the subsequent production of MSU crystals are known to activate the NLRP3 inflammasome, leading to inflammation [14]. Up until recently, soluble uric acid was considered inert and incapable of initiating inflammatory responses. However, Braga et al. demonstrated that soluble uric acid can activate the NLRP3 inflammasome through the production of mitochondrial reactive oxygen species [23]. Therefore, uric acid has been suggested as a danger-associated molecule or DAMP [24]. This paper sought to investigate the effect of soluble uric acid on the interplay between autophagy and the NLRP3 inflammasome during bacterial infection. We report that soluble uric acid enhanced autophagy flux and reduced the release of IL-1β. These findings suggest that uric acid may have a potential role in reducing septic inflammation through autophagy induction. Further investigation into this process may facilitate the discovery of new treatment regimens for gout patients.

## 2. Experimental Section

### 2.1. Reagents

Uric acid sodium salt, DMSO, Bovine Serum Albumin (BSA), paraformaldehyde, orthophosporic acid, and sulfuric acid (Sigma, St. Louis, MO, USA). Mouse and human IL-1β ELISA DuoSet kit (R&D Systems, Minneapolis, MN, USA). Autophagy Flux Detection kit (Abcam, Cambridge, UK). Liquicolor uric acid detection kit (EKF Diagnostics, Boerne, TX, USA). Rapamycin (Abcam, UK), Chloroquine (Abcam, UK). Dulbecco’s modified Eagle medium, RPMI1640, Fetal Bovine Serum (FBS), Penicillin/Streptomycin solution, 0.4% Trypan Blue (D-MEM) (Gibco via Thermo Fisher Scientific, Waltham, MA, USA). Nutrient agar, peptone, yeast extract, LB broth, and phosphate-buffered saline (Dulbecco A) tablets (Oxoid via Thermo Fisher, Waltham, MA, USA). Sulfonamide, N-(1-Naphthylethylenediamine) dihydride, and sodium nitrite (NaNO_2_) (Biochem Chemopharma, Loire, France). 4′,6-diamidino-2-phenylindole (DAPI) dilactate and propidium iodide (Invitrogen, Carlsbad, CA, USA).

### 2.2. Bacterial Culture

Three *Staphylococcus aureus* (*S. aureus*) strains and two *Klebsiella pneumoniae* (*K. pneumoniae*) strains were grown on nutrient agar plates and harvested after incubation at 37 °C for 24 h. Bacteria was fixed using 10% formalin solution as previously described [25]. The fixed bacteria were then washed thoroughly to remove traces of formalin, resuspended in phosphate buffered saline (PBS), then adjusted to optical density of 3.0 at 600 nm wavelength, and stored at 4 °C until further use. For macrophage infection assay using live bacteria, freshly grown *S. aureus* and *K. pneumoniae* were harvested and adjusted to optical density of 1.0 at 600 nm wavelength then diluted to optical density (OD) of 0.1 using antibiotic-free tissue culture media.

### 2.3. Uric Acid Preparation

Uric acid salt was dissolved in Dulbecco’s Modified Eagle Medium (DMEM) media and undissolved crystals were filtered using a 22 nm filter. The concentration of uric acid was measured following the manufacturer’s protocol for the uric acid detection kit. The concentration of uric acid was adjusted to approximately 19 mg/dL by adding fresh DMEM media.

### 2.4. Macrophage Induction Assay

To investigate autophagy induction in macrophages, the following cell lines were used: murine RAW264.7 macrophages that express uricase enzyme, LC3-GFP-tagged RAW264.7 murine macrophages that contain LC3, markers of autophagy activation, and human THP-1 macrophage-like monocytic cells that are devoid of uricase enzyme. Freshly grown RAW264 macrophages were harvested and the number of cells was adjusted to 5 × 10^5^ cells/mL with a viability above 85%. One ml of macrophages and 2 mL of fresh DMEM media were transferred into a coverslip containing 6-well tissue culture plate and allowed to grow and adhere overnight. The next day, designated wells of macrophages were divided into two groups, uric-acid-treated (UA) and non-treated cells. To mimic hyperuricemia, macrophages were incubated with uric-acid-containing media at (19 mg/dL). All wells were then stimulated with 150 µL of inactivated (formalin-fixed) bacterial suspension adjusted to an OD600 of 3.0. The dose of infection here is approximately at multiplicity of infection (MOI) of 50. Unstimulated cells in each plate were used as a negative control. Cells were incubated at 37 °C 5% (v/v) CO_2_ for 22–24 h. Supernatants were collected and stored at −20 °C. Additional media was removed and the cells were washed twice with PBS, fixed with 5% formalin, and stained with DAPI. LC3-GFP-tagged RAW264.7 were treated in a similar manner to RAW264.7 and used for confocal imaging. Further, culture media of freshly grown human THP-1 monocytes were replaced with uric-acid-containing DMEM media at a concentration of 19 mg/dL. Cell count was adjusted to 2.5 × 10^5^ cells/mL and transferred into 24-well tissue culture plates at 2 mL/well. THP-1 cells were then infected with 100 µL of inactivated (formalin-fixed) bacterial suspension and incubated at 37 °C 5% (v/v) CO_2_ overnight. Supernatants were collected and cells were resuspended and harvested for staining. Dose response experiments were performed using 96-well tissue culture plates. Briefly, RAW264.7 macrophages as well as THP-1 cell counts were adjusted to 1 × 10^6^ cells/mL and 250 µL of cell suspension transferred into each well. Cells were then stimulated with 50 µL of serially diluted bacterial inoculums adjusted at OD600 = 3.0 ranging from 100 to 5 µL.

### 2.5. Macrophage Infection with Live Bacteria

Freshly grown THP-1 and Murine RAW264.7 macrophages were harvested and cell counts adjusted to 1 million cell /mL in antibiotic-free RPMI 1640 and DEMEM, respectively, and transferred into 24-well tissue culture plates as described above. Macrophages were infected with live bacterial inoculum of *S. aureus* and *K. pneumoniae* at MOI of 10 in presence and absence of soluble uric acid (19 mg/dL) for three hours. Bacterial infection process was halted by the addition of the following antibiotics to each well: Gentamycin (10 µg/mL), Penicillin (50 I.U /mL), and Streptomycin (50 µg/mL). To assess the effect of autophagy induction on inflammasome activation and consequent IL-1β release, the autophagy chemical inducer Rapamycin was used at 7.5 µM final concentration in designated wells. In some experiments, Chloroquine (another chemical autophagy inducer) was used at 25 µM final concentration per well.

### 2.6. Autophagy Flux Measurement

RAW264.7 macrophages were infected with formalin-fixed bacteria in a coverslip containing 6-well plates and incubated overnight as mentioned above. Cells were stained using the autophagy flux detection kit (Abcam, UK) following the manufacture protocol. This autophagy kit contains a specific fluorescent probe that integrates with acidic autophagolyososmes and, therefore, measures active autophagic flux, not just arrested autophagolysosomes [19]. Stained cells on coverslips were placed on slides and viewed under confocal microscope (Olympus FLUOVIEW FV3000, Center Valley, PA, USA). Similarly, THP-1 monocytes were stained using the autophagy flux detection kit (Abcam, UK) and placed on slides for live imaging as previously described [19].

### 2.7. Confocal Microscopy Imaging and Image Analysis

Autophagy flux induction was assessed by taking three different images from random fields in each slide. Systemic image analysis was applied and the intensity of the green fluorescence and DAPI in each image was then analyzed using Image J software [26].

### 2.8. Human and Murine IL-1β Detection and Quantification

IL-1β measured in supernatants from induced THP-1cells and RAW264 macrophages using the DueSet human IL-1β and mouse IL-1β ELISA kit (R&D Systems, Minneapolis, MA, USA), respectively. TNF-α in supernatants from induced THP-monocytes was measured using the DueSet human TNF-α ELISA kit (R&D Systems, USA) as previously described [27].

### 2.9. Nitric Oxide Release Quantification

The Greiss reaction was used to quantitate the amount of nitric oxide released from murine RAW264 macrophages treated with or without uric acid and infected with formalin-fixed bacteria as previously described [28].

### 2.10. Statistical Analysis

Statistical analysis was performed using Microsoft Excel and GraphPad Prism software. Student *t*-test followed by One-way ANOVA analysis were conducted. *p* values less than 0.05 were considered significant.

## 3. Results

### 3.1. Soluble Uric Acid Modulates Autophagy Flux Induction in Macrophages during Bacterial Infection In Vitro

**Figure 1:**
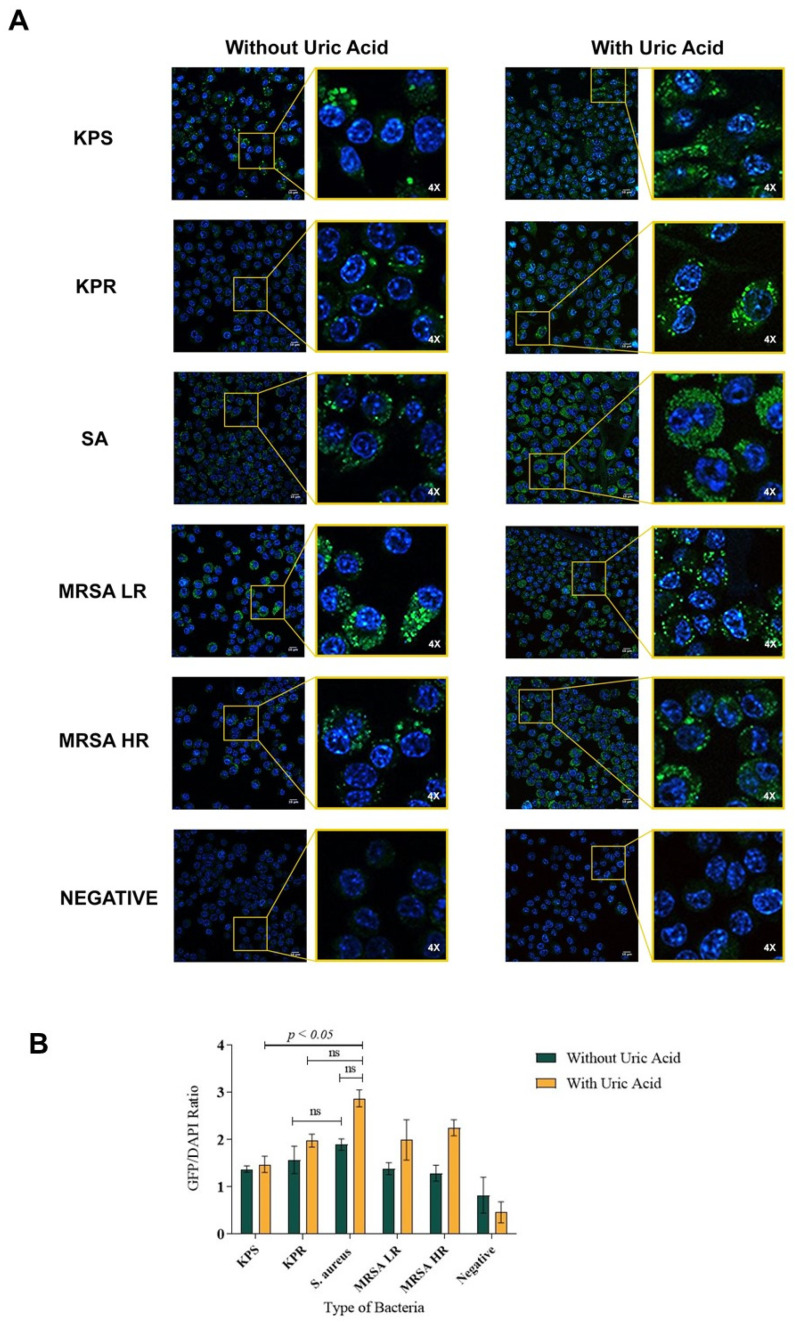
Soluble uric acid enhances autophagy flux in infected murine macrophages. Representative images of murine RAW264.7 macrophages infected with different strains of *Staphylococcus aureus* or *Klebsiella pneumoniae* and stained with autophagy flux detection kit. (**A**) Macrophages infected with bacteria only and macrophages infected with bacteria and soluble uric acid (19 mg/dL). DAPI-stained nuclei are shown in blue color, while active autophagy vacuoles are the green puncta. (**B**) Quantitative image analysis was performed using the intensity of GFP-to-DAPI ratio calculated in murine RAW264.7 macrophages induced by different strains of *S. aureus* and *K. pneumoniae* in presence and absence of uric acid stimulation (19 mg/dL). The inset is magnification of a representative image showing autophagy puncta (green) and nucleus DAPI staining (blue). The data represent the mean of three independent experiments with error bars depicting the standard error of the mean. The images were analyzed using ImageJ analysis software. *p* value < 0.05 are significant. KPS: *K. pneumoniae* sensitive to antibiotics; KPR: *K. pneumoniae* resistant to antibiotics; SA: *S. aureus*; MRSA LR: methicillin-resistant *S. aureus* with low level of resistance; MRSA HR: methicillin-resistant *S. aureus* with high level of resistance; UA: uric acid; ns: non-significant.

**Figure 2:**
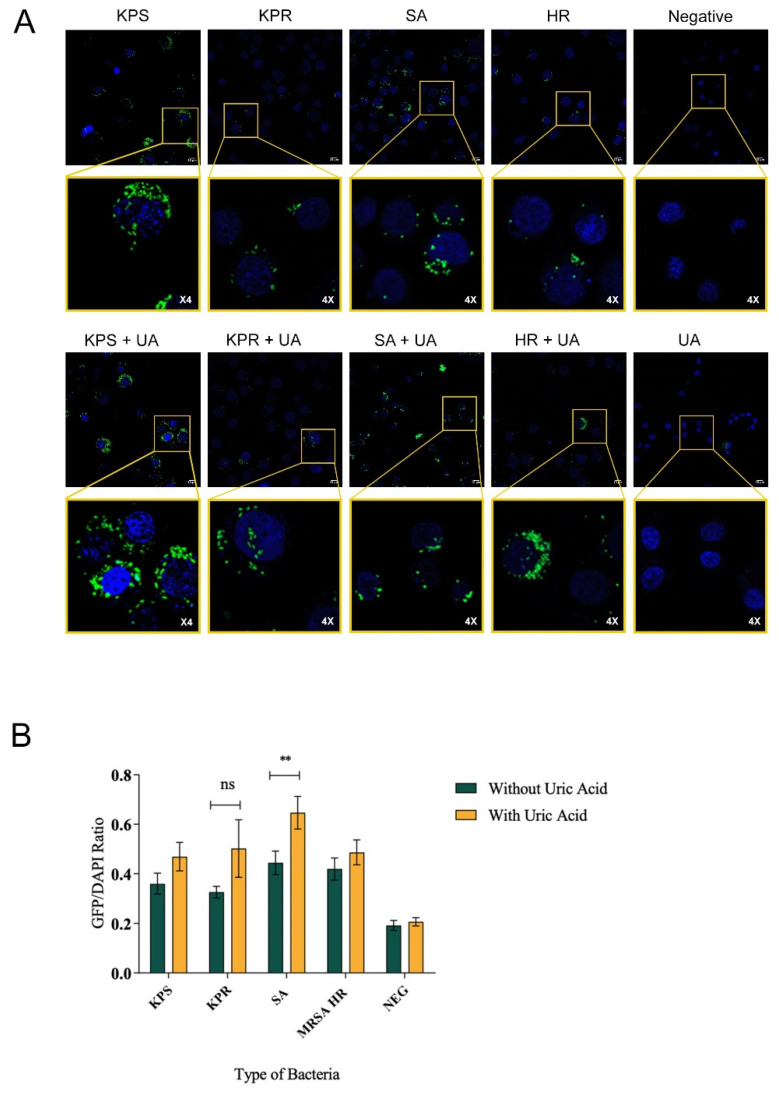
Soluble uric acid enhances autophagy flux in infected human monocytes. Representative images of human THP-1 monocytes induced with different strains of *S. aureus* or *K. pneumoniae* and stained using the autophagy flux detection kit. (**A**) THP-1 monocytes induced with bacteria only or THP-1 monocytes induced with bacteria and soluble uric acid (19 mg/dL). DAPI-stained nuclei are shown in blue color, while active autophagy vacuoles are the green puncta. The inset is magnification of a representative image showing autophagy puncta (green) and nucleus DAPI staining (blue). (**B**) Quantitative image analysis of autophagy flux in human THP-1 monocytes. Autophagy flux quantitation was performed by calculating the ratio of GFP-to-DAPI intensity observed in stained THP-1 monocytes. The data represent the mean of three independent experiments with error bars depicting the standard error of the mean. The images were analyzed using ImageJ analysis software. *p* value < 0.05 are significant. KPS: *K. pneumoniae* sensitive to antibiotics; KPR: *K. pneumoniae* resistant to antibiotics; SA: *S. aureus*; MRSA HR: methicillin-resistant *S. aureus* with high level of resistance; UA: uric acid; ns: non-significant. ** *p* value < 0.05 for SA with uric acid in comparison to SA without uric acid.

Autophagy flux was investigated in macrophages infected with clinically relevant bacterial strains of *Klebsiella pneumonia* and *Staphylococcus aureus*, representing Gram-negative and Gram-positive bacterial infections, respectively, in presence and absence of soluble uric acid. In order to eliminate interference from various bacterial virulence factors, inactivated formalin-fixed bacteria were used to infect macrophages. Here, we report that bacterial infection induced autophagy flux in murine RAW264.7 macrophages induced with Gram-positive or Gram-negative bacteria compared to non-infected macrophages (Figure 1A). We also report that uric acid enhanced autophagy flux in infected RAW264.7 macrophages specifically when *Staphylococcus aureus* strain was used (Figure 1A). Basal level autophagy flux was also monitored in uninfected macrophages, which were used as a control. Uric acid alone without bacterial trigger did not induce autophagy flux in murine macrophages (Figure 1A). In this study, five different clinical bacterial strains were used in these experiments. To ensure accurate data comparison, autophagy flux was quantitated using systemic image analysis ImageJ software (Figure 1B). Similar results were observed in human THP-1 monocytes when infected with bacteria compared to non-infected cells in presence or absence of soluble uric acid (19 mg/dL) (Figure 2A,B). In order to confirm active autophagy induction in macrophages, LC3-GFP-tagged RAW264.7 macrophages were used, infected with bacteria in presence and absence of uric acid and autophagy inducers Rapamycin and Chloroquine. Autophagy induction was measured by the fluorescence of GFP puncta formation reflecting the aggregation of LC3, the marker of autophagy, and the conversion of LC3 form to autophagy membrane-bound LC3II form. (Supplementary Figures S1 and S2). Taken together, the data show that soluble uric-acid-enhanced autophagy flux in murine macrophages and human monocytes during bacterial infections.

### 3.2. Soluble Uric Acid Decreases IL-1β Production in Murine Macrophages and Human Monocytes during Bacterial Infection In Vitro

**Figure 3:**
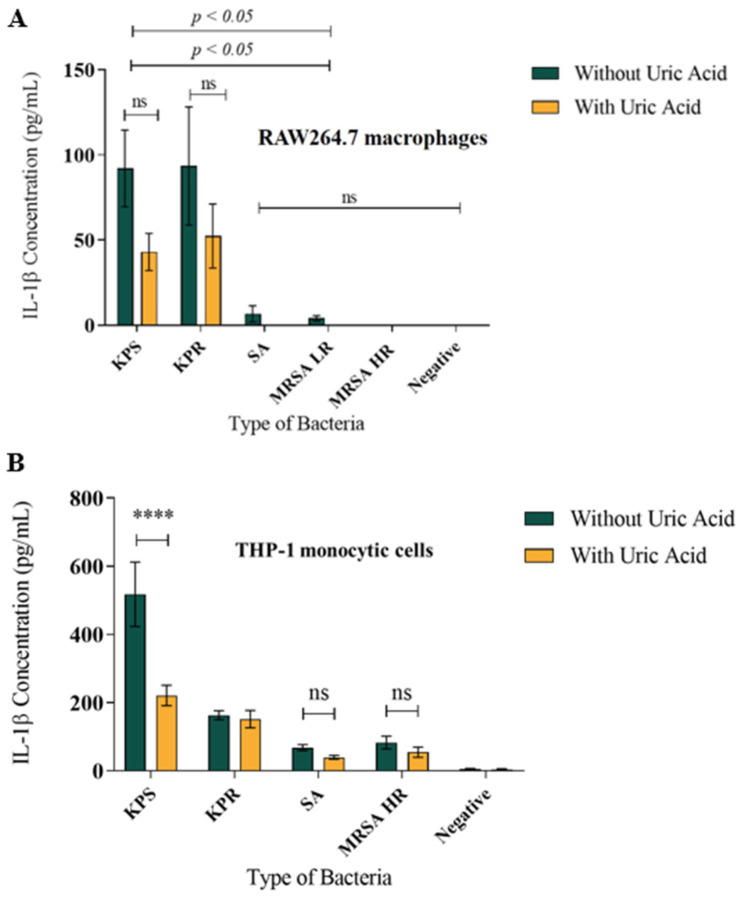
Soluble uric acid attenuates IL-1β release from infected macrophages. (**A**) Murine RAW264.7 macrophages infected with formalin-fixed bacteria in presence and absence of soluble uric acid (19 mg/dL) and incubated overnight. IL-1β release in the supernatants was quantitated using ELISA method. These data represent the mean of six independent experiments with bars showing the standard error of the mean. (**B**) IL-1β released from human THP-1 monocytic cells induced similar to panel A above. KPS: *K. pneumoniae* sensitive to antibiotics; KPR: *K. pneumoniae* resistant to antibiotics; SA: *S. aureus*; MRSA LR: methicillin-resistant *S. aureus* with low level of resistance; MRSA HR: methicillin-resistant *S. aureus* with high level of resistance; UA: uric acid; ns: not significant. *p* value < 0.05 are significant; **** in comparison to KPS without uric acid.

Here, we report that uric acid decreased IL-1β release from infected murine RAW264.7 macrophages (Figure 3A) as well as from human THP-1 monocytes (Figure 3B) when compared to non-infected cells. Soluble uric acid alone did not induce IL-1β release nor unstimulated RAW264.7 macrophages or THP-1 cells. We observed a significant difference in the amount of IL-1β release from stimulated macrophages when induced with Gram-negative bacteria compared to Gram-positive bacteria. Infection with *Klebsiella pneumoniae* lead to significantly larger amounts of IL-1β release from both murine and human macrophages when compared to *Staphylococcus aureus* strains, in presence and absence of uric acid (Figure 3A,B). IL-1β release was dose-dependent and similar results were obtained where *K. pneumoniae* strains induced significantly higher amounts of IL-1β release compared to *S. aureus* strains (Supplementary Figure S3).

**Figure 4:**
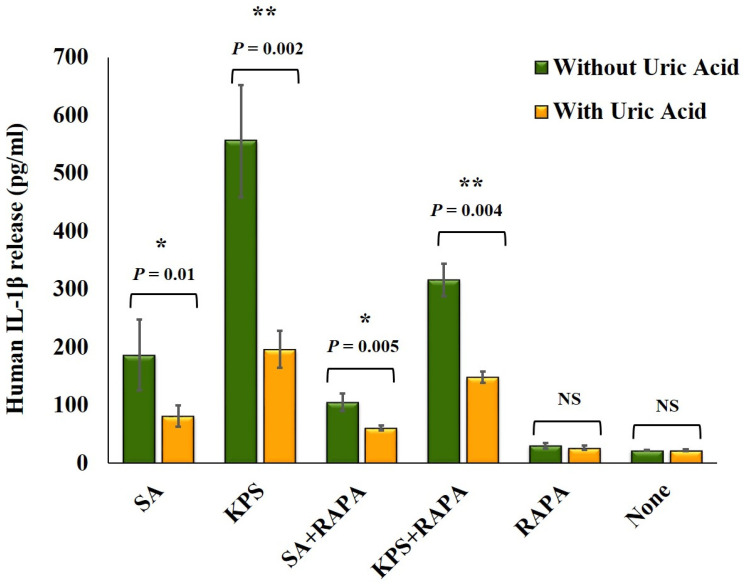
Soluble uric acid attenuates IL-1β released from macrophages infected with live bacteria. IL-1β release from human THP-1 monocytic cells infected with live bacteria at a multiplicity of infection (MOI) of 10 in presence and absence of soluble uric acid (19 mg/dL) and Rapamycin for three hours prior to the addition of Gentamycin, Penicillin, and Streptomycin, then incubated overnight. IL-1β released in the supernatants was quantitated using ELISA method. These data represent the mean of two independent experiments with bars showing the standard error of the mean. SA: *S. aureus*; KPS: *K. pneumoniae* sensitive to antibiotics; RAPA: Rapamycin; UA: uric acid; ns: not significant. *p* value < 0.05 are significant, * in comparison to SA without Rapamycin, ** in comparison to KPS without Rapamycin.

To confirm the effect of soluble uric acid on modulating IL-1β release, we infected THP-1 monocytic cells and murine RAW264.7 macrophages using live bacterial strains *S. aureus* (SA) and *K. pneumoniae* (KPS). We observed that soluble uric acid significantly reduced IL-1β release from THP-1 cells (Figure 4) infected with live either SA or KPS strains. Further, reduction in the amounts of IL-1β released was observed in the presence of the autophagy inducer Rapamycin (Figure 4). Similar results were observed when RAW264.7 macrophages were infected with live bacteria in presence and absence of soluble uric acid and Rapamycin.

### 3.3. Soluble Uric Acid Does Not Affect TNF-α Release from Human Monocytes during Bacterial Infection

**Figure 5:**
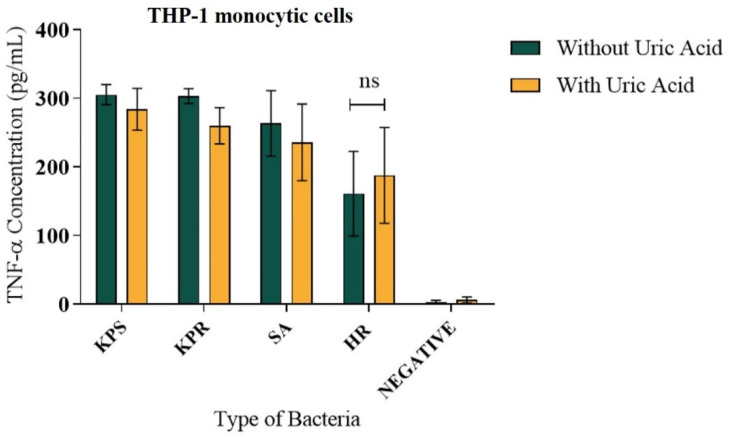
TNFα release from infected human THP-1 monocytes in presence of soluble uric acid. Human THP-1 monocytic cells were infected with formalin-fixed bacteria in presence and absence of soluble uric acid (19 mg/dL) and incubated overnight. IL-1β release in the supernatants was quantitated using ELISA method. These data represent the mean of six independent experiments with bars showing the standard error of the mean. KPS: *K. pneumoniae* sensitive; KPR: *K. pneumoniae* resistant; SA: *S. aureus*; MRSA HR: methicillin-resistant *S. aureus* with high resistance; UA: uric acid; ns: not significant.

In contrast to the pattern of IL-1β release, no significant difference was observed in the pattern of TNF-α release from THP-1 cells induced with *K. pneumonia* strains compared to *Staphylococcus aureus* strains in presence or absence of soluble uric acid in vitro (Figure 5). Soluble uric acid alone did not induce TNF-α release nor unstimulated THP-1 macrophages (Figure 5). TNF-α release pattern was consistent when THP-1 cells were induced in a dose-dependent manner (Supplementary Figure S4). Since IL-1β release requires inflammasome activation, the data suggest that soluble uric acid effect on *K. pneumonia* infection is inflammasome-mediated. On the contrary, soluble uric acid effect during *Staphylococcus aureus* infection induced larger autophagy flux in macrophages but significantly less IL-1β release. Taken together, the data reflect the cross talk between autophagy and inflammasome and demonstrate the potential antioxidant effect of soluble uric acid on autophagy pathway in vitro.

### 3.4. Soluble Uric Acid Enhances Nitric Oxide Production in Murine Macrophages during Bacterial Infection

**Figure 6:**
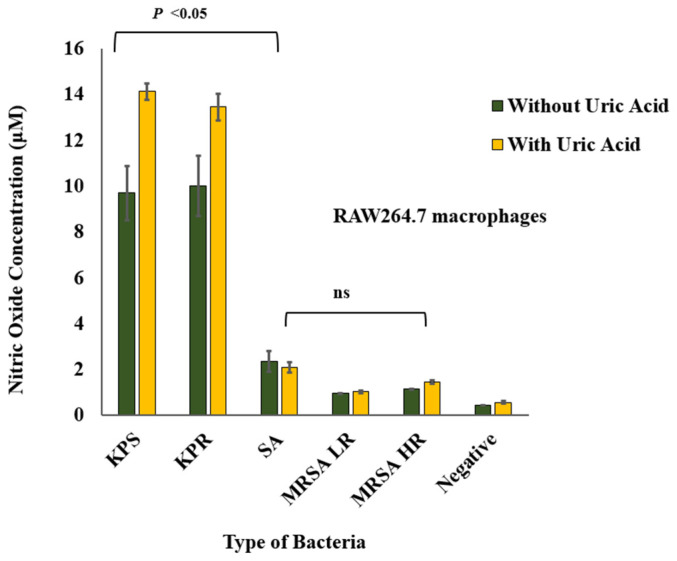
Nitric oxide release from infected murine macrophages in presence and absence of uric acid. Murine RAW264.7 macrophages were infected with formalin-fixed bacteria in presence and absence of soluble uric acid (19 mg/dL) and incubated overnight. Nitric oxide release in the supernatants was quantitated using the Griess method. These data represent the mean of six independent experiments with bars showing the standard error of the mean. KPS: *K. pneumoniae* sensitive to antibiotics; KPR: *K. pneumoniae* resistant to antibiotics; SA: *S. aureus*; MRSA LR: methicillin-resistant *S. aureus* with low level of resistance; MRSA HR: methicillin-resistant *S. aureus* with high level of resistance; UA: uric acid; ns: not significant. *p* value < 0.05 are significant.

Soluble uric acid potentiated nitric oxide release from RAW264.7 macrophage infected with *K. pneumoniae* strains when compared to *S. aureus* strains (Figure 6). Similar to the IL-1β release pattern, *K. pneumoniae* strains induced significantly larger amounts of nitric oxide when compared to *S. aureus* strains in vitro. Soluble uric acid alone did not induce nitric oxide release nor unstimulated RAW264.7 macrophages. A dose-dependent response to infection was also observed and this striking difference between *K. pneumoniae* and *S. aureus* was also maintained (Supplementary Figure S5).

In order to validate the results of autophagy flux modulation during bacterial infection, the effect of soluble uric acid on lipopolysaccharide (LPS)-stimulated macrophages was also investigated. Soluble uric acid alone did not induce autophagy flux nor proinflammatory cytokine release. Although autophagy flux was observed when macrophages were stimulated with highly purified *E. coli* LPS (10 ng/ml) (Supplementary Figure S6A), as expected, a significant release of IL-1β and nitric oxide (Supplementary Figure S6B), and TNFα, was observed (data not shown). As a control for autophagy flux, RAW264.7 macrophages were treated with the autophagy inducer Rapamycin (Supplementary Figure S6C).

## 4. Discussion

In this study, we examined the dynamics of autophagy and inflammation-mediated responses to bacterial infections in presence of soluble uric acid using both murine RAW264.7 macrophages that naturally express the uricase enzyme and human THP-1 monocytes that naturally lack uricase. Uricase is an enzyme that breaks down insoluble uric acid to allantoin in most mammals other than humans and apes. Humans and apes possess an inactive form of the uricase. Increased levels of uric acid are thought to have provided a survival advantage, which led to loss of the uricase activity during human evolution [8]. Although high levels of uric provided an evolutionary advantage, it predisposed today’s humans to dire consequences of hyperuricemia [29]. The effect of soluble uric acid was not influenced by the presence or absence of uricase in this in vitro cellular induction model. We were able to see a similar pattern in both human and murine cell lines.

It is becoming widely accepted that *S. aureus* can infect non-phagocytic cells and act as an intracellular pathogen. It is hypothesized that S. aureus increases autophagy but halts autophagy flux in non-phagocytic host cells to facilitate its survival [30,31]. In addition, in phagocytic cells such as macrophages, phagocytosis as well as autophagy are both enhanced after *S. aureus* infection [32]. When *S. aureus* bacteria is phagocytosed by the host cells, toxins produced by the bacteria (mainly α-toxin) disintegrate the phagosomal membrane. Autophagy is activated in an attempt to contain the cellular damage [33]. However, it is worth it to mention that *S. aureus* strains that do not disrupt the phagosomal membrane do not enhance autophagy in infected macrophages [34]. There are no studies on the effect of formalin-fixed or heat-killed inactivated *S. aureus* on the process of autophagy. Therefore, it is likely that the high autophagy flux induced with *S. aureus* strains observed in our study is a result of toxins inactivation that remained enclosed inside the bacteria and not secreted after formalin fixation. Moreover, since the bacteria were inactivated, their host-defense-evading mechanisms were defective, autophagy was not halted, and active autophagy flux was also increased as observed. In our study, we observed higher levels of IL-1β release from THP-1 cells and RAW264.7 when live S. aureus was used in an in vitro infection assay (Figure 4).

Regarding autophagy induction by Gram-negative bacteria, few studies investigated autophagy induction during *K. pneumoniae* infections. A study reported that ATG7-mediated autophagy appears to play a critical role in the clearance of *K. pneumoniae* from alveolar macrophages [35]. In our study, LPS-alone and heat-killed *K. pneumoniae* demonstrated an increase in autophagy, indicating that cellular or membrane components in live bacteria are the driving forces for autophagy activation [36]. We found that both *S. aureus* and *K. pneumoniae* stimulate autophagy flux. However, induction with formalin-fixed *S. aureus* resulted in higher autophagy activation compared to *K. pneumoniae*. This difference could be attributed to different types of toxins produced by the two types of bacteria and their effect on the phagosomal membrane since one is a model of Gram-positive bacteria while the other represents Gram-negative bacteria. Nevertheless, infection with live bacteria to better mimic the effect of these two bacterial types on autophagy during infection in macrophages is warranted.

The interplay between soluble uric acid and autophagy in macrophages is not fully understood. Recently, Crişan et al. reported that priming human macrophages with soluble uric acid led to the inhibition of autophagy accompanied by high levels of IL-1 β. Lack of autophagy and increase in proinflammatory IL-1β worsen the outcomes of subsequent exposure to an inflammatory stimuli in gout patients [37]. On the other hand, studies on kidney cell lines PTECs [38] and PC12 [39] found that uric acid enhanced autophagy. It can be deduced that uric acid exhibits paradoxical effects on autophagy depending on how and when uric acid is used for stimulation. We demonstrated that co-induction of macrophages with soluble uric acid and bacteria induced autophagy flux in murine and human cells lines in vitro (Figure 1 and Figure 2, Figures S1 and S2). This could be a result of the simultaneous stimulation of macrophages with both uric acid and bacteria, likely overwhelming the cells. In addition, we noticed a significant difference in the increase of autophagy flux during *S. aureus* versus *K. pneumonia* infection (Figure 1 and Figure 2). This is likely due to the intrinsic differences between Gram-negative and Gram-positive bacteria. A study comparing *S. aureus* and *E. coli* infections demonstrated that IL-1β released during *S. aureus* was less when compared to *E. coli* infections [40].

Soluble uric acid activates the NLRP3 inflammasome, allowing the production of IL-1β [23]. The successful release of IL-1β requires two signals. The first is a “priming” signal induced by PAMPs (pathogen-associated molecular patterns) or DAMPs (danger-associated molecular patterns). Priming leads to the expression of pro-IL-1β, the inactive precursor of IL-1β. A second signal then activates the NLRP3 inflammasome, which in turn activates caspase-1, ultimately cleaving generating mature IL-1β [41]. It has been shown that high levels of IL-1β levels induce autophagy as a protective mechanism to prevent exaggerated inflammatory responses [21]. These observations are alien to our findings. In addition to enhanced autophagy flux, co-stimulation with bacteria and uric acid led to decreased levels of IL-1β released by macrophages (Figure 3). The control of IL-1β production by autophagy could be exerted in several ways. For example, pro-IL-1β could be directly degraded by autophagy [21], or by degrading damaged organelles and preventing the release of danger signals [42]. It is worth it to note that when uric acid was the sole stimulator, it inhibited autophagy in murine macrophages, and had no visible effect on autophagy in human monocytes. We attribute this disparity to the presence of uricase in murine macrophages but not human monocytes. However, further work is need to confirm this hypothesis.

TNF-α is an inflammatory cytokine with a wide range of functions. It is implicated in protection and/or exacerbation of a wide range of diseases, inflammatory conditions, and infections [43]. TNFα release is not inflammasome-mediated; thus, no difference was observed on the amount of TNF-α released in presence or absence of uric acid during bacterial infections. However, uric acid alone did not induce an inflammatory response that lead to the release of TNF-α, suggesting that uric-acid-mediated inflammation does not involve TNF-α (Figure 4).

We also observed higher levels of nitric oxide released during co-induction of murine macrophages with soluble uric acid and *K. pneumoniae* when compared to soluble uric acid and *S. aureus* strains co-induction (Figure 5). Nitric oxide is a reactive nitrogen species that acts as a signaling molecule. It is an abundant cellular messenger found throughout the body [44]. The interplay between nitric oxide and autophagy is a complex process [45]. This is due to the paradoxical relationship between nitric oxide and autophagy, in which the literature shows both inhibitory and stimulatory effect of nitric oxide on autophagy [8,46,47,48]. The contradictory effect of nitric oxide on autophagy suggests the presence of a feedback loop between autophagy and nitric oxide that remains to be elucidated. While nitric oxide was shown to have an inhibitory effect on the NLRP3 inflammasome [49], we observed that high levels of nitric oxide and IL-1β coincided during co-induction with soluble uric acid and *K. pneumoniae* and the opposite was observed during uric acid and *S. aureus* co-infection (Figure 5).

In summary, this study highlights the basics of a complex cross-talk between autophagy and inflammation during bacterial infection and uric acid stimulation. However, live bacterial infection is needed to confirm and further support the results obtained. Moreover, although we used soluble uric acid in our experiments, microcrystals of uric acid, which appear as fine short needles, are not always visible using light microscopy and might have been present and could be responsible for some of the results we observed.

## 5. Conclusions

The interplay between autophagy and the inflammasome during sterile and septic inflammation is a complex process yielding various cellular outcomes. Hyperuricemia is associated with pathological consequences. Our study reports that soluble uric acid modulated autophagy flux induction in macrophages infected with bacteria, leading to the suppression of the inflammatory cytokine IL-1β. More studies are warranted to further understand the intricate interactions between autophagy and the inflammasome during sterile and septic inflammatory conditions. Understanding the signaling cross-talk between autophagy and the inflammasome during hyperuricemia will shed light on the mechanisms involved in sudden gout attacks and facilitate therapeutic target discovery and design.

## Supplementary Materials

The following are available online at https://www.mdpi.com/2227-9059/8/12/598/s1, Figure S1: Soluble uric acid attenuates IL-1β release from infected human THP-1 cells in a dose dependent manner. Figure S2: TNFα release from infected human THP-1 cells in a dose dependent manner. Figure S3: Nitric oxide release from infected murine RAW264.7 macrophages in a dose dependent manner. Figure S4: Autophagy flux induced by LPS in murine macrophages.
