# Supplementary material for: The Role of Soluble Uric Acid in Modulating Autophagy Flux and Inflammasome Activation during Bacterial Infection in Macrophages
Source: Biomedicines. 2020 Dec 12;8(12):598. doi: 10.3390/biomedicines8120598 (PMC7763195; doi:10.3390/biomedicines8120598)
Supplement: Supplementary file 1 [file biomedicines-08-00598-s001.pdf]

Supplementary Materials

The Role of Soluble Uric Acid in Modulating Autophagy Flux and Inflammasome Activation during Bacterial Infection in Macrophages

Duha Al-Awad[1#], Nada Al-Emadi[1#], Marawan Abu Madi[1], Asmaa A. Al-Thani[1,2,4], Susu M. Zughaier[3,4*]

[1]Biomedical Science Department, College of Health Sciences, [2]Biomedical Research Center, and [3]College of Medicine, [4]Biomedical and Pharmaceutical research Unit, QU Health, Qatar University, Doha, Qatar, P.O. Box 2713

#Joint first author. * szughaier@qu.edu.qa

Supplementary Figures:

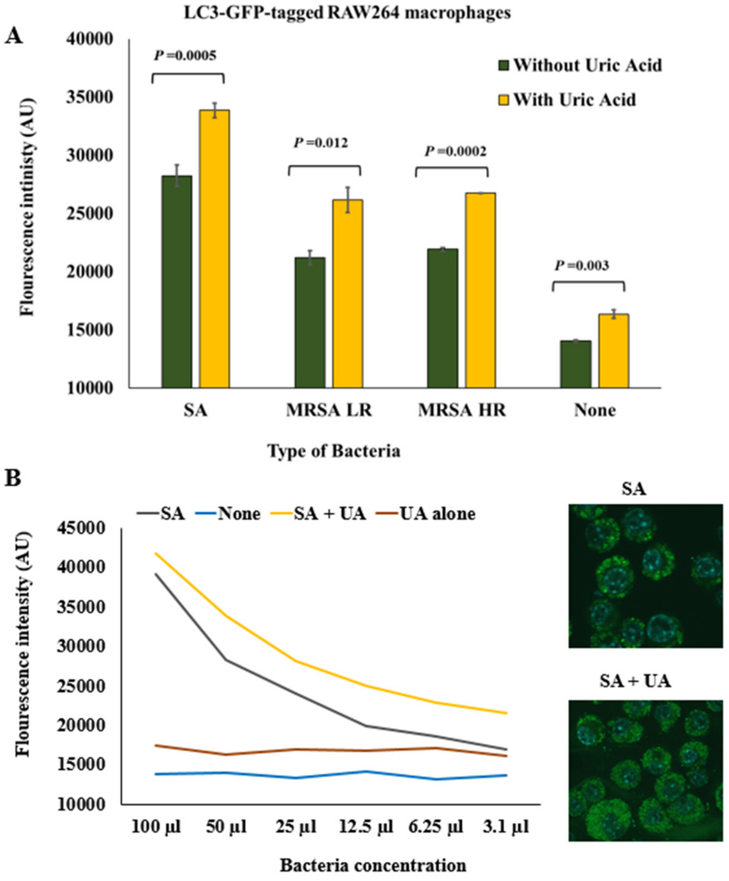

Supplement Figure S1: Soluble uric acid enhances autophagy induction in LC3-GFP-tagged RAW264.7 macrophages in a dose dependent manner. LC3-GFP-tagged RAW264 macrophages infected with formalin-fixed *Staphylococcus aureus* (SA) bacteria **A**: 50 µl (MOI~ 18) of suspension OD600 = 3.0, in presence and absence of soluble uric acid (19mg/dL) and incubated overnight. **B**: LC3-GFP-tagged RAW264.7 macrophages infected in a dose-dependent manner. Autophagy induction was measured by LC3-GFP fluorescence intensity. SA: S. aureus; MRSA LR: methicillin resistant S. aureus with low level of resistance; MRSA HR: methicillin resistant S. aureus with high level of resistance; UA: Uric Acid.

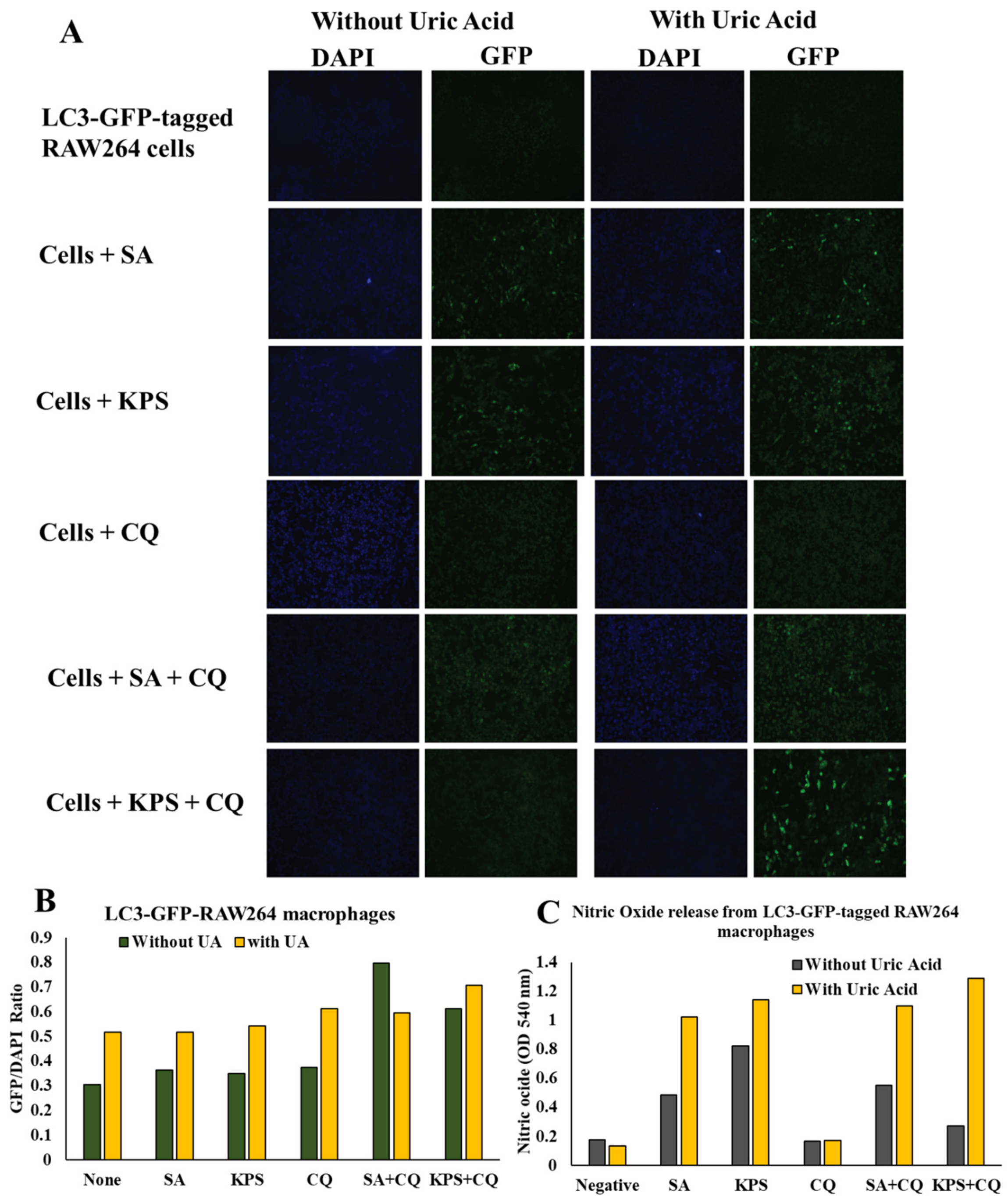

Supplement Figure S2: Soluble uric acid enhances autophagy induction in LC3-GFP-tagged RAW264 macrophages. A: Representative images of autophagy induction in LC3-GFP-tagged RAW264.7 macrophages infected with formalin-fixed *Staphylococcus aureus* (SA) or *K. pneumoniae* (KPS) in presence and absence of uric acid stimulation (19 mg/dL) and the autophagy inducer Chloroquine (CQ, 60nM) and stained with DAPI. **B**: Quantitative image analysis was performed using the intensity of GFP to DAPI ratio calculated in LC3-GFP-tagged RAW264.7 macrophages. **C**: Nitric oxide release from LC3-GFP-tagged RAW264.7 macrophages induced in panel A.

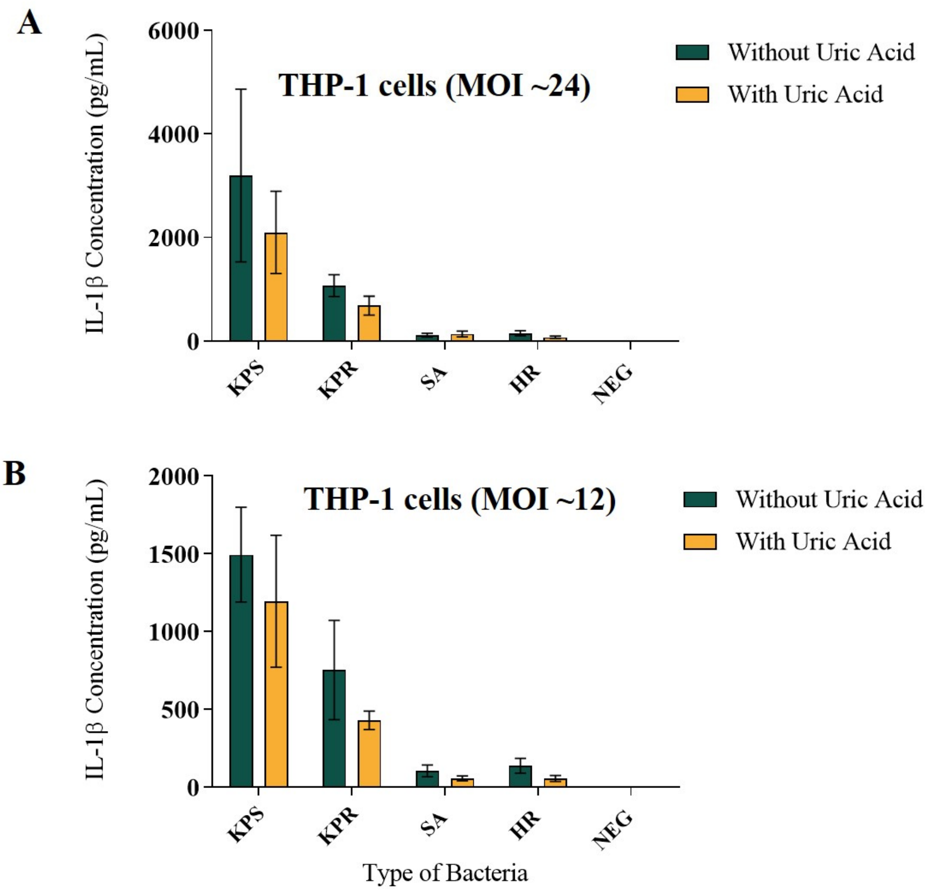

Supplement Figure S3: Soluble uric acid attenuates IL-1β release from infected human THP-1 cells in a dose dependent manner. THP-1 cells infected with formalin-fixed bacteria **A**: 70 μl (MOI~ 24) and **B**: 35 μl (MOI~ 12) of suspension OD600 = 3.0, in presence and absence of soluble uric acid (19mg/dL) and incubated overnight. IL-1β release in the supernatants was quantitated using ELISA method. This data represent the mean of six independent experiments with bars showing the standard error of the mean. KPS: *K. pneumoniae* sensitive to antibiotics; KPR: *K. pneumoniae* resistant to antibiotics; SA: *S. aureus*; MRSA HR: methicillin resistant *S. aureus* with high level of resistance; UA: Uric Acid; NS: not significant. p value < 0.05 are significant.

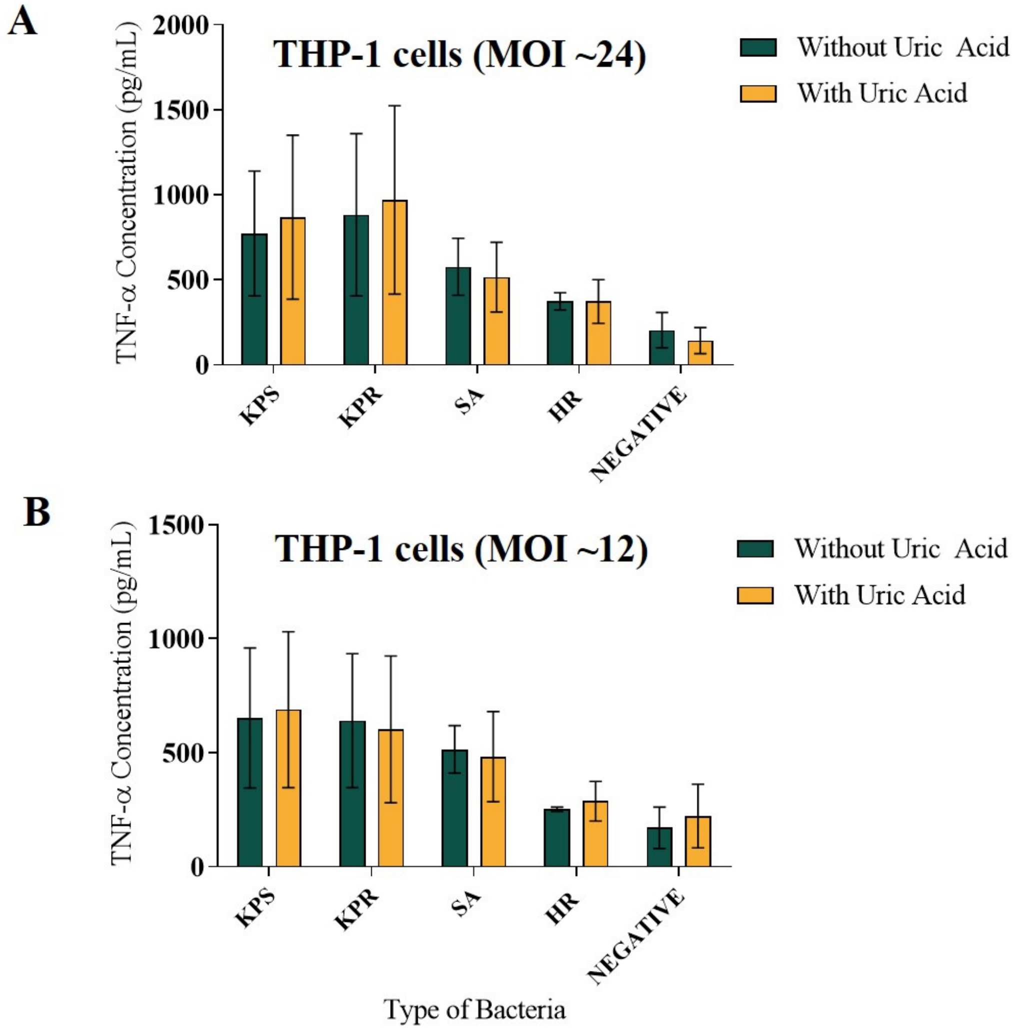

Supplement Figure S4: TNFα release from infected human THP-1 cells in a dose dependent manner. THP-1 cells infected with formalin-fixed bacteria **A**: 70 µl (MOI~ 24) and **B**: 35 µl (MOI~ 12) of suspension OD600 = 3.0, in presence and absence of soluble uric acid (19mg/dL) and incubated overnight. TNFα release in the supernatants was quantitated using ELISA method. This data represent the mean of six independent experiments with bars showing the standard error of the mean. KPS: *K. pneumoniae* sensitive to antibiotics; KPR: *K. pneumoniae* resistant to antibiotics; SA: *S. aureus*; MRSA HR: methicillin resistant *S. aureus* with high level of resistance; UA: Uric Acid; NS: not significant. p value < 0.05 are significant.

A

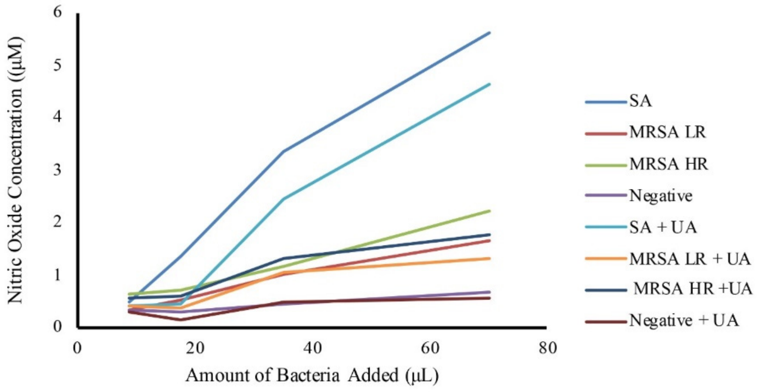

B

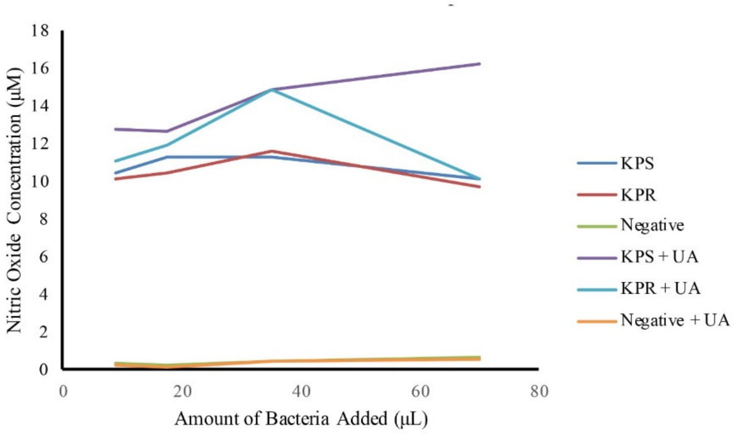

Supplement Figure S5: Nitric oxide release from infected murine RAW264.7 macrophages in a dose dependent manner. RAW264.7 macrophages infected with various doses of formalin-fixed *S. aureus* strains (**A)** and *K. pneumoniae* (**B)** from bacterial suspension OD600 = 3.0, in presence and absence of soluble uric acid (19mg/dL) and incubated overnight. Nitric oxide release was quantified using the Griess method. This data represent the mean of six independent experiments with bars showing the standard error of the mean. KPS: *K. pneumoniae* sensitive to antibiotics; KPR: *K. pneumoniae* resistant to antibiotics; SA: *S. aureus*; MRSA LR: methicillin resistant *S. aureus* with low level of resistance; MRSA HR: methicillin resistant *S. aureus* with high level of resistance; UA: Uric Acid.

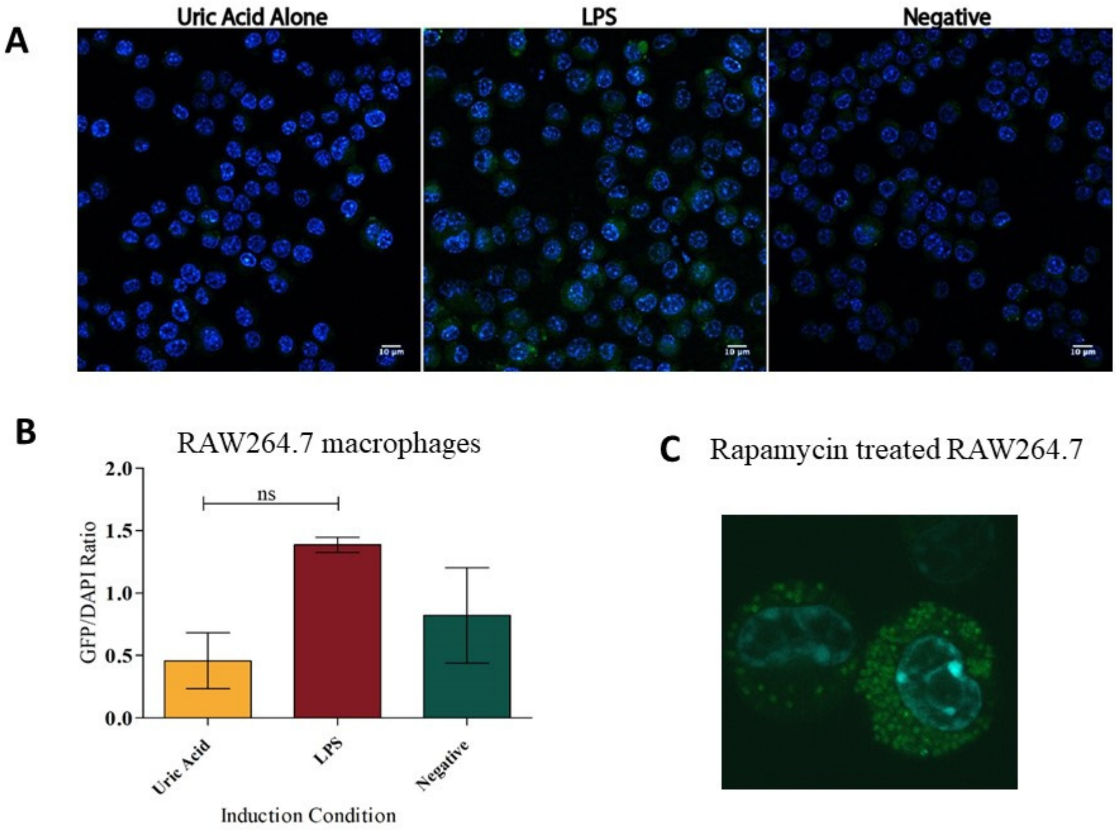

Supplement Figure S6: **Autophagy flux induced by LPS in murine macrophages. A**: Representative images of murine RAW264.7 macrophages stimulated with *E. coli* LPS (10ng/ml) or soluble uric acid (19 mg/dL). DAPI stained nuclei are shown in blue color, while active autophagy vacuoles are the green puncta. **B**: Quantitative image analysis was performed using the intensity of GFP to DAPI ratio calculated in murine RAW264.7 macrophages. The data represent the mean of three independent experiments with error bars depicting the standard error of the mean. The images were analyzed using ImageJ analysis software. p value < 0.05 are significant. C: Autophagy flux in RAW264.7 macrophages treated with 7.5 μM Rapamycin.
